# Identification and validation of FaP1D7, a putative marker associated with the biosynthesis of methyl butanoate in cultivated strawberry (*Fragaria x ananassa*)

**DOI:** 10.1038/s41598-017-17448-1

**Published:** 2017-12-12

**Authors:** Mian Chee Gor, Chrishani Candappa, Thishakya de Silva, Nitin Mantri, Edwin Pang

**Affiliations:** 1 School of Science, RMIT University, Plenty Road, PO Box 71, Bundoora, Victoria, 3083 Australia; 2 Griffith Institute for Drug Discovery (GRIDD), Don Young Road, Nathan, Queensland 4122 Australia

## Abstract

Breeding strawberry (*Fragaria x ananassa*) with enhanced fruit flavour is one of the top breeding goals of many strawberry-producing countries. Although several genes involved in the biosynthetic pathways of key aroma compounds have been identified, the development and application of molecular markers associated with fruit flavour remain limited. This study aims to identify molecular markers closely linked to genes controlling strawberry aroma. A purpose-built Subtracted Diversity Array (SDA) known as *Fragaria* Discovery Panel (FDP) was used for marker screening. Polymorphic sequences associated with key aroma compounds were identified from two DNA bulks with extreme phenotypes, established using 50 F_1_ progeny plants derived from Juliette X 07-102-41 cross, two strawberry genotypes differing in aroma profile. A total of 49 polymorphic markers for eight key aroma compounds were detected using genotypic data of the extreme DNA bulks and phenotypic data obtained from gas chromatography-mass spectrometry (GC-MS). A similarity search against the physical maps of *Fragaria vesca* revealed that FaP1D7 is linked to genes potentially involved in the synthesis of methyl butanoate. A C/T SNP was detected within the feature, which could possibly be converted to a molecular tool for rapid screening of the strawberry accessions for their methyl butanoate production capacity.

## Introduction

Some studies suggest that the flavour quality of many fruits including strawberry has deteriorated due to conventional breeding practices^1,2^. Consumers nowadays are also increasingly discriminating and demand highly flavourful fruits. Hence, the development of cultivars with improved flavour is becoming one of the main breeding priorities for many strawberry-producing countries. Strawberry aroma has been studied extensively in the past. Over 300 compounds corresponding to a complex mixture of esters, furanones, aldehydes, alcohols, terpenes, lactones and sulphur compounds have been identified^3–8^. Of these, only about 20 main volatile compounds have been determined to dominate the typical strawberry aroma based on sensory descriptive analysis and their odour activity values^9^. For instance, the key esters including methyl butanoate, methyl hexanoate, ethyl butanoate and ethyl hexanoate have been attributed to the fruity notes whereas the aldehydes such as (*E*)-hex-2-enal and hexanal account for the green and grassy notes in strawberry aroma. Furaneol and mesifuranne have been described as the two most important compounds contributing to the sweet and caramel-like flavour in ripe strawberry fruits^9–11^. Apart from chemical compositions, sweetness intensity and fruit firmness also have effects on sensory perception. A previous study demonstrated correlation between sucrose concentration and total volatiles, indicating the dependence of secondary metabolism to primary metabolism during fruit ripening developmental stage. The authors also presented some specific sugar-independent volatiles capable of enhancing the sweetness intensity of strawberry fruits^12^.

In recent years, strawberry flavour research has progressed from chemical and sensory analyses to the investigation of biosynthesis and genetic control of important aroma compounds^13^. Genes involved in the biosynthesis of esters and terpenes, the *SAAT* (strawberry-specific alcohol acyl transferase) and *FaNES1* (*Fragaria x ananassa* nerolidol synthase 1) respectively, have been identified using cDNA microarray technology^14,15^. Chambers, *et al*.^16^ reported the presence/absence of a *FaNES1* allele that could mostly predict the linalool-producing and non-producing cultivated and wild strawberry materials, with a few exceptions. Moreover, *FaOMT* (*Fragaria x ananassa O*-methyltransferase) and *FaQR* (*Fragaria x ananassa* quinone oxidoreductase) genes were also reported to be involved in the formation of mesifuranne and furaneol, respectively^17–19^. Zorrilla-Fontanesi, *et al*.^20^ showed that a 30-bp indel in the promoter region of the *FaOMT* allele is responsible for the high expression of mesifuranne content and may be turned into an important tool for strawberry breeding. Additionally, two recent studies have shown that a fatty acid desaturase *FaFAD1* gene was correlated with the production of γ-decalactone in strawberry fruit^21^ and a PCR-based marker co-segregating with the phenotype was developed^22^.

Despite the development of PCR-based markers for linalool and γ-decalactone, breeding for improved strawberry flavour has been relatively slow due to the complex genetic control of fruit flavour biogenesis. Therefore, it is crucial to identify genomic regions corresponding to other key aroma compounds for the selection of elite plants towards marker-assisted breeding. We recently developed a Subtracted Diversity Array (SDA)^23^ called *Fragaria* Discovery Panel (FDP) as a platform for screening molecular markers associated with agronomically important traits^24^. We combined the application of FDP with Bulked Segregant Analysis (BSA)^25^, a rapid method for detecting DNA markers linked to any specific gene in the genome. Taking advantage of the discriminatory power of SDA for genotyping closely related species within the same genus^26–28^, we have successfully identified a putative marker, FaP2E11 possibly involved in controlling day-neutrality in octoploid strawberry^24^. Here, we extended the utility of the subtracted gDNA microarray-assisted BSA for the identification of polymorphic markers linked to the loci determining strawberry aroma.

## Results and Discussion

### Variability and distribution of aroma compounds in the parental genotypes and F_1_ segregating population

Aroma profiling of the fruits from the F_1_ population derived from a cross between Juliette and 07-102-41 allowed identification of 41 aroma compounds from the headspace of strawberry puree. Of these, 23 compounds (Table 1) were described as aroma-active compounds in strawberry based on gas chromatography quantification, their odour activity values (OAVs) and sensory analysis^9,10^. The remaining 18 aroma compounds, namely, ethyl propanoate, 4-methyl-2-pentanone, ethyl isobutanoate, methyl 2-methylbutanoate, ethyl tiglate, isobutyl butanoate, isopentyl 3-methylbutanoate, methyl octanoate, ethyl benzoate, (*E*)-hex-3-enyl butanoate, (*Z*)-hex-3-enyl butanoate, trans-2-hexenyl butanoate, ethyl octanoate, 3-methylbutyl hexanoate, hexyl hexanoate, (*E*)-cinnamyl acetate, and (*Z*)-ethyl cinnamate and benzaldehyde) exhibited a population mean value lower than 0.1% and were detected infrequently in the F_1_ population. Some of these compounds have been reported to have low broad-sense heritability values in strawberry^29^. Hence, they were excluded for DNA marker development as environmental factors may have a greater effect in controlling the inheritance of these compounds.

**Table 1:** Descriptive statistics for 23 volatile compounds analysed in the F_1_ population and in the parental genotypes ‘07-102-41’ and ‘Juliette’.

Aroma compounds	F_1_ population			07-102-41		Juliette		*t*-Test (for parents)		
	Mean^a^	Min^b^	Max^b^	Mean^c^	SD^c^	Mean^c^	SD^c^	*t* value^d^	*df*	*p* ^d^
**Esters**										
Methyl butanote	9.4	0.0	34.3	15.4	6.1	0.0	0.0	4.36	2.0	*
Methyl 3-methylbutanote	0.3	0.0	2.7	0.0	0.0	0.0	0.0	—	—	—
Ethyl butanote	4.8	0.0	23.2	7.2	1.9	3.2	1.4	3.38	5	*
Isopropyl butanote	0.5	0.0	3.1	0.0	0.0	0.0	0.0	—	—	—
Ethyl 2-methylbutanote	0.2	0.0	2.0	0.0	0.0	1.5	0.1	−12.49	5	**
Ethyl isovalerate	0.7	0.0	14.5	0.0	0.0	1.8	0.1	−10.48	5	**
Isoamyl acetate	0.8	0.0	7.3	0.0	0.0	0.0	0.0	—	—	—
Methyl hexanoate	14.1	0.0	48.1	25.0	1.6	0.0	0.0	26.98	2.0	**
Butyl butanoate	0.25	0.0	1.9	3.7	1.2	0.0	0.0	5.57	2.0	*
Ethyl hexanoate	6.9	0.0	35.6	16.2	1.3	10.1	1.2	6.05	5	**
(*Z*)-Hex-3-enyl acetate	0.7	0.0	5.0	0.0	0.0	0.7	1.0	-42.51	5	**
Hexyl acetate	2.1	0.0	35.8	0.0	0.0	1.5	0.3	−2.41	5	ns
(E)-Hex-2-enyl acetate	2.5	0.0	14.8	0.0	0.0	2.5	2.7	−1.73	3.0	ns
Isopropyl hexanoate	0.2	0.0	1.2	0.0	0.0	0.0	0.0	—	—	—
Benzyl acetate	1.4	0.0	23.7	0.0	0.0	0.0	0.0	—	—	—
Hexyl butanoate	0.4	0.0	9.2	0.0	0.0	0.0	0.0	—	—	—
**Aldehydes**										
Hexanal	1.0	0.0	15.2	0.0	0.0	0.0	0.0	—	—	—
(*E*)-Hex-2-enal	7.5	0.0	63.7	0.0	0.0	0.0	0.0	—	—	—
**Furanone**										
Mesifuranne	3.2	0.0	32.4	0.0	0.0	5.7	3.1	−4.43	3.0	*
**Terpenes**										
Linalool	3.3	0.0	18.3	9.1	0.8	6.2	1.1	9.95	5	*
(*E*)-Nerolidol	7.7	0.0	36.9	17.4	4.1	60.6	6.4	−11.23	5	**
α-Terpineol	0.9	0.0	10.0	0.0	0.0	0.0	0.0	—	—	—
**Lactones**										
γ-Dodecalactone	1.4	0.0	7.9	0.0	0.0	4.8	1.8	−6.55	3.0	**

All values are normalised peak areas in relative composition (%).

^a^Mean of all analysed F_1_ individuals, based on three technical replicates per genotype.

^b^F_1_ individuals with the lowest (Min) and highest (Max) relative composition, mean from three technical replicates.

^c^Mean and standard deviation (SD) from all technical replicates.

^d^ *t*-Test between the parents ‘07-102-41’ and ‘Juliette’.

ns: not significant (*p* > 0.05); *Significant at 0.05 ≥ *p* > 0.01; **Significant at 0.01 ≥ *p* > 0.001.

Table 1 shows the descriptive statistics of the 23 volatile compounds from the F_1_ population compared to the parental genotypes. Overall, 14 compounds were detected in either one or both of the parents whereas nine compounds were not identified in any of the parental genotypes but were detected in the F_1_ plants. Distinctive aroma patterns were observed between parents, with ‘07-102-41’ displaying higher levels of C4 and C6 esters and linalool while the levels of C7 and C8 esters, mesifuranne, (*E*)-nerolidol and γ-dodecalactone were higher in ‘Juliette’ (Table 1). This result is in agreement with the study which purported that strawberry aroma patterns (i.e., the combination and intensity of aroma compounds) were genotype-dependent^30^. Furthermore, most of the compounds detected in both parents showed a significant difference in the levels of production except for hexyl acetate and (*E*)-hex-2-enyl acetate. Nonetheless, diversity in the relative composition could be inferred from a range between minimum and maximum values of the F_1_ population (Table 1).

**Figure 1:**
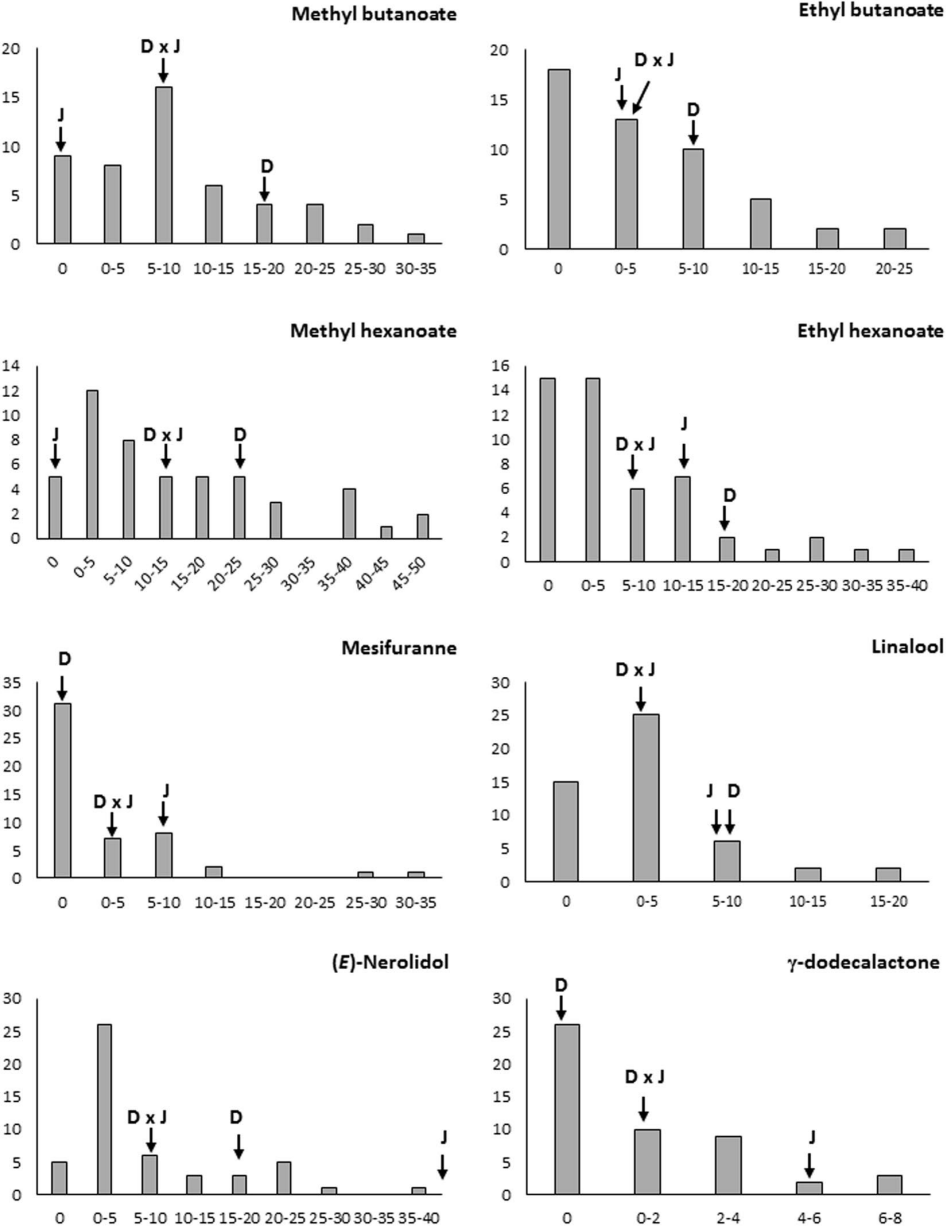
Frequency distribution of key aroma compounds measured as relative peak areas in the ‘07-102-41’ x ‘Juliette’ progeny. The mean values of the parents and F_1_ population are indicated by arrows (D: 07-102-41; J: Juliette; D x J, respectively). *x*-axis: relative composition (%), *y*-axis: the number of individual plants.

Out of the 23 aroma compounds, we focused our analysis on eight compounds which were considered to have the greatest impact on strawberry aroma due to their low threshold values and high OAVs^9,10^. The frequency distributions of these eight key aroma compounds, including four esters (methyl butanoate, ethyl butanoate, methyl hexanoate, ethyl hexanoate), one furanone (mesifuranne), two terpenes (linalool and (*E*)-nerolidol) and one lactone (γ-dodecalactone) are illustrated in Fig. 1. Most of the frequency distributions are highly or moderately skewed towards lower values or zero except for methyl hexanoate which approached a bimodal distribution. This result is in accordance with the frequency distributions for aroma compounds analysed in other fruit crops such as strawberry^31^; apple^32^ and peach^33^ except that none of the compounds found in this study have a frequency distribution skewed towards the higher values. This type of frequency distribution is typical of a trait with polygenic inheritance, indicating that aroma compound production is under the control of more than one gene^32^. In addition, transgressive segregation, the phenomenon where the individuals in a segregating population exhibit phenotypes that are extreme compared to the parental genotypes^34–36^, was observed for all the compounds assessed except for (*E*)-nerolidol (Fig. 1). This phenomena could be due to the formation of new combination of alleles at multiple loci underlying quantitative trait differences between the two parents^37,38^. The high levels of transgressive segregation may be due to the significant differences between parental means (Table 1), that is, the parental genotypes were phenotypically very different from each other^39^.

Overall, the eight key aroma compounds were selected for DNA marker discovery based on: (1) their importance in the characterisation of strawberry aroma^9,10,30^ and (2) their significant differences in parental means (Table 1). In theory, the probability of detecting the alleles controlling the trait of interest is higher if the parental means are significantly different from each other. Furthermore, the aroma compositions of two Australian-grown cultivars, Albion and Juliette, have been evaluated extensively over ten weeks in one growing season. The inheritance analysis revealed that methyl butanote, ethyl butanoate, mesifuranne and (*E*)-nerolidol possessed high broad sense heritability values (46.0–74.4%)^29^. This result coincides with the genotype-by-environment (G × E) analysis, where all selected compounds, except linalool, were found to be predominantly influenced by genotype. These findings also suggest that the yearly phenotypic changes of strawberry plants were not as great as seasonal variations^40^ and thus, the greater probability to identify molecular markers associated with the selected key aroma compounds. Although no F_2_ population was available for analysis, it is plausible that the F_1_ individuals with extreme phenotypes (i.e. much higher or not-detected) can be used as DNA materials in the subsequent Bulked Segregant Analysis (BSA) for the discovery of alleles segregating for the key aroma compounds.

### Identification of polymorphic loci associated with key aroma compounds

Signal intensities generated from the hybridisation of the ‘H’ and ‘L’ bulks onto the FDP were used to identify polymorphic loci associated with key aroma compounds. The genotype data was subjected to Discriminant Function Analysis (DFA) using stepwise method to select the most discriminating markers from the original 287 features. It was further used to reduce the number of polymorphic markers and form classification models up to four markers (Table 2). Interestingly, FaP1E7 was detected in more than two key aroma compounds, i.e. methyl butanoate (referred to FaP1E7MB) and methyl hexanoate (FaP1E7MH). The classification of the original cases in the training set based on the discriminant functions showed that only ethyl butanote, mesifuranne and γ-dodecalactone were correctly classified (100%) into the two phenotypic extreme groups. For the other key aroma compounds, the proportion of correctly classified original cases ranged from 83.3% to 95.8% (Table 2). In addition, the accuracy of group membership prediction for the test set by cross-validation ranged from 79.2–100% for the key aroma compounds assessed (Table 2). The high percentage of correct classification implies that all the features selected by DFA are good predictors for the levels of key aroma compound biosynthesis in strawberry.

**Table 2:** Putative DNA markers selected by DFA, the classification results for the original cases and the estimated proportion for any future dataset.

Key volatile compounds	DFA-selected markers^*^	Classification Results (%)	
		Original	Cross-validation
Methyl butanoate	**FaP1E7MB**, FaP1D7	95.8	95.8
Ethyl butanoate	FaP1D11, FaP3B9, FaP3A2	100.0	95.8
Methyl hexanoate	FaP2A11, **FaP1E7MH**	87.5	87.5
Ethyl hexanoate	FaP1B3, FaP1G2,	95.8	95.8
Mesifuranne	FaP2G4, FaP2E6, FaP3H11	100.0	100.0
Linalool	FaP3E12	83.3	79.2
(*E*)-Nerolidol	FaP2D11, FaP1G8, FaP3F10	95.8	95.8
γ-Dodecalactone	FaP2E1, FaP1A7, FaP3F8, FaP3E8	100.0	87.5

^*^Features in bold represent putative DNA markers that were detected in more than two key volatile compounds.

A similar approach was employed in a previous study which identified AFLP markers associated with stress tolerance index in Sardari wheat ecotypes, where stepwise analysis was firstly used to identify the number of polymorphic markers and then further applied to reduce the number of markers and form classification models of up to 24 markers^41^. Our results are in accordance with several previous studies, where the rate of misclassification increased as the number of predictors decreased^41–43^. Alwala^42^ showed that only 61.7–62.2% of correct classification was obtained based on five markers and a minimum of 10 markers were needed to achieve more than 90% of correct classification. The low cross-validation error rate produced in this study implies that the reduced sets of putative DNA markers selected by the DFA possessed high predictive power, hence, a stronger association between the marker and the phenotype^43^.

The DFA-selected features were validated using Fisher’s ratio to measure the linear discriminating power of the features. These features were arranged in decreasing order of Fisher’s ratio and only the top 10 features are presented in Table 3. Out of the 20 DFA-selected features, only six (FaP1D7, FaP1D11, FaP1B3, FaP3E12, FaP2D11, and FaP1A7) features showed high Fisher’s ratio values. The other DFA-selected putative markers displayed low Fisher’s ratio values and therefore, were excluded from further statistical analysis. In general, a larger Fisher’s ratio value indicates greater differences between the means of two extreme bulks. Hence, the features with very strong signal intensities in the ‘H’ DNA bulk but very weak signal intensities in the ‘L’ DNA bulk, or vice versa, will potentially generate larger Fisher’s ratio values provided that the background noise inherent in the microarray system is minimum (sum of the variances of the two extreme bulks). Hence, the putative DNA markers with higher Fisher’s ratio could possibly be the best in discriminating between the two phenotypic extreme bulks. The subsequent Independent Samples *t*-Test showed that the mean differences between the ‘H’ and ‘L’ DNA bulks for eleven putative DNA markers (FaP1D7, FaP1D11, FaP3A2, FaP3B9, FaP1E7MH, FaP1B3, FaP1G2, FaP2E6, FaP3E12, FaP2D11, and FaP1A7) selected by DFA were statistically significant (*p* < 0.01) (Table 4). This result is also corroborated by six of the features with high Fisher’s ratio (FaP1D7, FaP1D11, FaP1B3, FaP3E12, FaP2D11, and FaP1A7) as putative DNA markers which could be the predictor for their respective key aroma compounds.

**Table 3:** List of top 10 features ranked in decreasing order based on their respective Fisher’s ratio for key volatile compounds.

Fisher’s ratio	Key Volatile Compounds							
Ranking	Methyl butanoate	Ethyl butanoate	Methyl hexanoate	Ethyl hexanoate	Mesifuranne	Linalool	(*E*)-Nerolidol	γ-Dodecalactone
1	FaP1C8	FaP3C12	FaP3H11	FaP2B5	FaP1H10	FaP2F6	FaP2D4	FaP1A7^*^
2	FaP1A4	FaP4D1	FaP4B10	FaP2B8	FaP2G5	FaP1D1	FaP2E3	FaP1G9
3	FaP1A10	FaP1F12	FaP4C4	FaP1B3^*^	FaP2E2	FaP1C11	FaP2B1	FaP1H3
4	FaP1E11	FaP3E8	FaP3C12	FaP4B3	FaP3F8	FaP4A8	FaP2A12	FaP1G8
5	FaP1D7^*^	FaP2C2	FaP3E11	FaP2E3	FaP1H5	FaP3E11	FaP1B3	FaP1D1
6	FaP1F1	FaP1D11^*^	FaP4D6	FaP2B1	FaP4C11	FaP1B3	FaP2D11^*^	FaP4C11
7	FaP1E1	FaP4C4	FaP3F4	FaP1D7	FaP2F3	FaP3E12^*^	FaP1B5	FaP4C8
8	FaP2D7	FaP3C7	FaP2H3	FaP3G10	FaP1H8	FaP2F12	FaP2D9	FaP1G6
9	FaP2F4	FaP1C6	FaP4A3	FaP2C6	FaP1E11	FaP1C1	FaP1D1	FaP4B6
10	FaP2B5	FaP2F8	FaP3D10	FaP3F4	FaP1D5	FaP2B3	FaP3F2	FaP4D4

^*^Putative DNA markers which were also selected by DFA.

**Table 4:** Group statistics and Independent Samples *t*-Test for the putative DNA markers selected by DFA.

Key volatile compounds	DFA-selected markers	H		L		*t*-Test (for two bulks)		
		Mean	SD	Mean	SD	*t* value	*df*	*p*
Methyl butanoate	FaP1D7^b^	248.23	84.05	578.61	291.75	−3.77	12.8	**
	FaP1E7MB^a^	242.69	91.59	192.24	97.77	1.31	22	ns
Ethyl butanoate	FaP1D11^b^	279.03	85.56	1333.86	541.59	−6.66	11.6	**
	FaP3A2	81.80	16.68	180.47	72.65	−4.59	12.2	**
	FaP3B9	17.72	4.95	35.11	16.73	−3.45	12.9	**
Methyl hexanoate	FaP1E7MH^a^	13.48	6.31	136.56	95.59	−4.45	11.1	**
	FaP2A11	96.35	48.97	151.74	111.30	−1.58	15.1	ns
Ethyl hexanoate	FaP1B3^b^	44.43	14.18	142.83	35.33	−8.95	14.5	**
	FaP1G2	340.87	113.77	640.48	293.72	−3.30	14.2	**
Mesifuranne	FaP2E6	90.28	26.69	18.70	8.90	8.81	13.4	**
	FaP2G4	78.51	53.40	64.81	18.80	0.84	22	ns
	FaP3H11	34.96	12.62	37.67	15.79	−0.47	22	ns
Linalool	FaP3E12^b^	884.13	521.74	226.77	218.23	4.03	14.7	**
(*E*)-Nerolidol	FaP1G8	684.26	421.21	807.65	535.49	−0.63	22	ns
	FaP2D11^b^	493.63	156.16	303.39	130.25	3.24	22	**
	FaP3F10	448.95	276.61	274.31	155.03	1.91	17.3	ns
γ-Dodecalactone	FaP1A7^b^	282.66	119.63	106.71	57.26	4.60	22	**
	FaP2E1	1451.21	887.38	795.11	989.51	1.71	22	ns
	FaP3E8	317.99	214.62	314.93	224.38	0.03	22	ns
	FaP3F8	175.01	132.88	223.85	468.06	−0.35	22	ns

^a^Putative DNA markers which were selected for more than two key volatile compounds.

^b^Putative DNA markers which displayed high Fisher’s ratio values.

SD Standard deviation.

**Significant at *p* < 0.01 ns: Not significant (*p* > 0.05).

**Figure 2:**
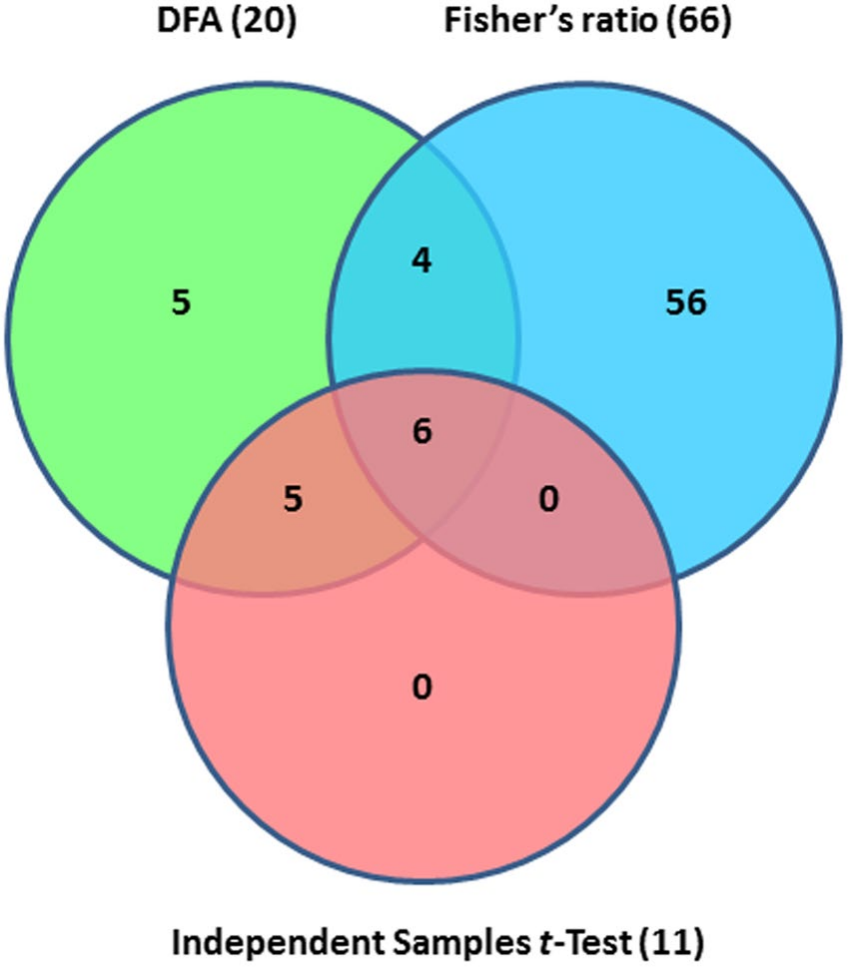
Venn diagram analysis of *Fragaria* Discovery Panel features selected by three statistical analyses. A three-way Venn diagram showing the putative DNA markers in the intersection of DFA (green), Fisher’s ratio (top 10 features; blue) and Independent Samples *t*-Test (*p* < 0.01; red) for all key aroma compounds assessed.

The three-way Venn diagram revealed six features at the intersection of the three statistical analyses (Fig. 2). Among the six features, FaP1D7, FaP1D11, and FaP1B3 were found to be putatively associated with ester compounds, i.e. methyl butanoate, ethyl butanoate and ethyl hexanoate, respectively. Additionally, FaP3E12 and FaP2D11 were putatively correlated with linalool and (*E*)-nerolidol, respectively whereas FaP1A7 was putatively associated with γ-dodecalactone. In general, features exhibiting significant differences of group means are expected to yield a higher Fisher’s ratio value. However, the intersection between DFA and Independent Samples *t*-Test revealed five other features (FaP3A2, FaP3B9, FaP1E7MH, FaP1G2, and Fa2E6) that differed significantly in their group means were not included in the top 10 features with the highest Fisher’s ratios (Fig. 2). Their low Fisher’s ratio values may be explained by smaller differences between group means or the greater sum of the variances of the two extreme bulks. Some of these features (FaP3A2, FaP3B9, FaP1E7MH, and FaP2E6) displayed low signal intensities as their group means were relatively lower compared to the other features (Table 4). In contrast, the Fisher’s ratio value of FaP1G2 that showed high signal intensities was lowered by a large sum of the variances (data not shown). Moreover, the group means of nine other features (FaP1E7MB, FaP2A11, FaP2G4, FaP3H11, FaP1G8, FaP3F10, FaP2E1, FaP3E8 and FaP3F8) were statistically not significant (*p* > 0.01). This result further supports the data obtained from the Fisher’s ratio analysis, where all these features have low Fisher’s ratio values (Table 4).

### Sequence identity and position of putative molecular markers

The identities of the six putative markers were revealed by DNA sequencing and similarity search against the *Fragaria vesca* draft genome (v1.1) using the PFR and GDR strawberry servers (GDR, 2009; PFR, 2010). Out of the six features sequenced, FaP1A7 and FaP1D7 appeared to be nuclear-specific whereas FaP1B3, FaP1D11, FaP2D11 and FaP3E12 were chloroplast-specific (Table 5). The nuclear-specific features did not a match any genes in the *F. vesca* genome database, indicating that they belong to the non-coding DNA region possibly associated to the genes involved in the biosynthesis of aroma compounds. The full sequence of FaP1A7 matched perfectly (100% E-value: 0.0) to a DNA region on linkage group 6 (LG6:21708323..21708764, scf0513196:589686..590127) whereas FaP1D7 was highly similar (E-value: 1e^−38^) to a genomic region on linkage group 2 (LG2:17544790..17544932, scf0513123:85522. 85664). For chloroplast-specific features, FaP1B3 showed significant similarity (E-value: 5e^−45^) to gene32946, a chloroplastic-like NAD(P)H-quinone oxidoreductase subunit H located on the scf0510865:52.396. The FaP1D11 was highly similar (E-value: e^−133^) to gene32967, a chloroplastic-like ATP synthase subunit alpha positioned on the scf0510833:190.1040. In contrast, 77% of FaP2D11 and the full sequence of FaP3E12 matched completely (E-value: 0.0) to scf0510759:1.513 and scf0513205:141.680, respectively. These two features did not correspond to any genes in the chloroplast genome (Table 5).

**Table 5:** Putative identity of the most discriminatory features searched against the *Fragaria vesca* draft genome (v1.1). E-value regarded as significant if <1e^−5^.

Clones	Length (bp)	Landmark or region	Sequence description	E-value	Specific to target
FaP1A7	442	LG6:21708323.21708764, scf0513196:589686-590127	Genomic DNA region on linkage group 6	0.0	γ-Dodecalactone
FaP1B3	343	gene32946 on scf0510865:52..396	NAD(P)H-quinone oxidoreductase subunit H, chloroplastic (similar to)	5e^−45^	Ethyl hexanoate
FaP1D7	627	LG2:17544790..17544932, scf0513123:85522.. 85664	Genomic DNA region on linkage group 2	1e^−38^	Methyl butanoate
FaP1D11	850	gene32967 on scf0510833:190..1040	ATP synthase subunit alpha, chloroplastic (similar to)	1e^−133^	Ethyl butanoate
FaP2D11	670	scf0510759:1..513	N/A	0.0	(*E*)-Nerolidol
FaP3E12	539	scf0513205:141..680	N/A	0.0	Linalool

**Figure 3:**
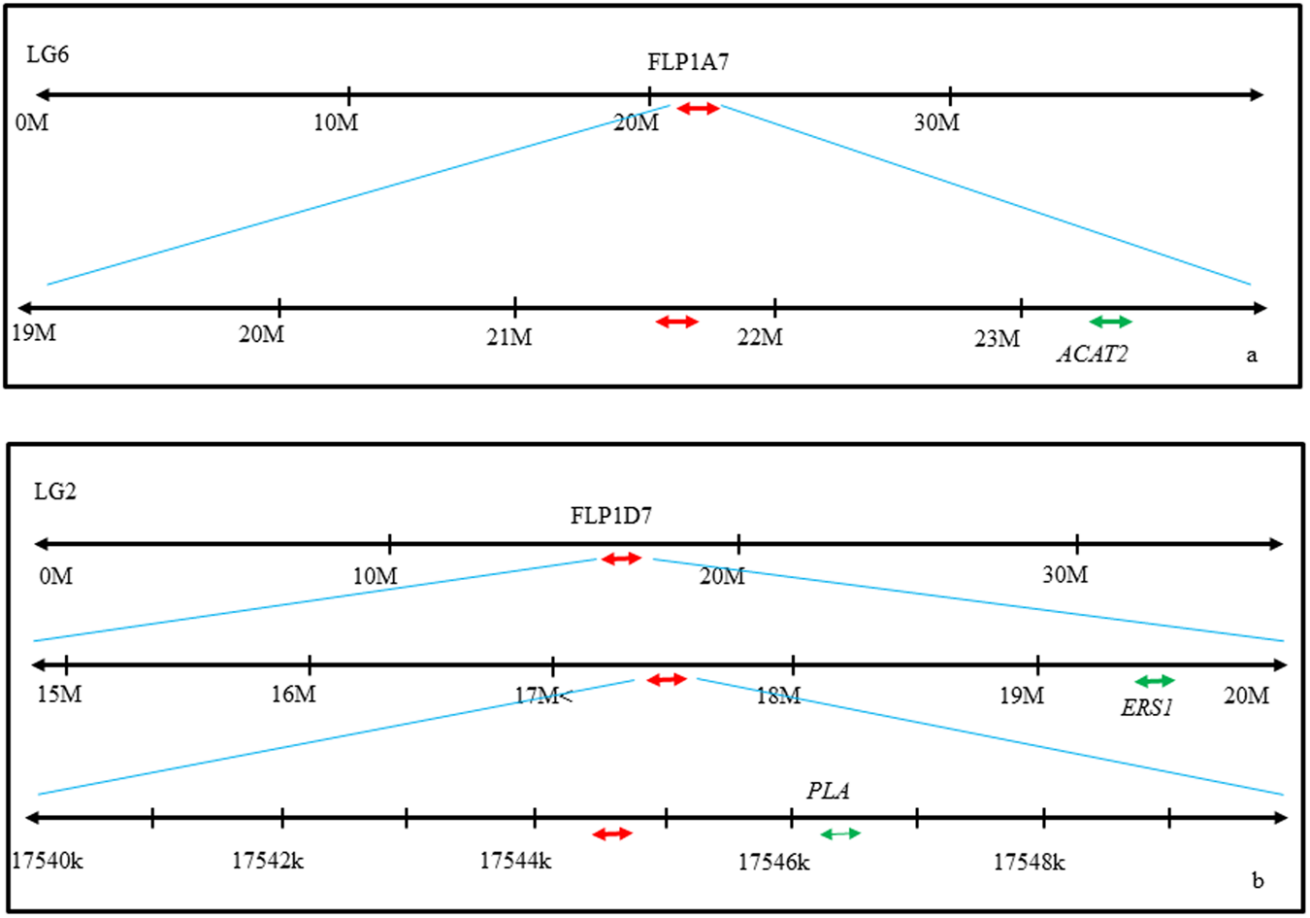
Landmark of selected putative DNA markers (red arrows) mapped onto the *F. vesca* draft genome (v1.1) and location of genes associated with key volatile compounds (green arrows). (**a**) FaP1A7 on LG6:21708323..21708764. *ACAT2*: acetyl-CoA acetyltransferase (cytosolic). (**b**) FaP1D7 on LG2:17544790..17544932. *PLA*: Patatin-related phospholipase A; *Ers1*: Ethylene receptor.

To identify genes possibly involved in the production of key volatile compounds, the genes situated within 5 cM on either side of the nuclear-specific features (FaP1A7 and FaP1D7) were manually searched on the same linkage group using the PFR Strawberry Server (PFR, 2010). Figure 3a shows a gene corresponding to *Arabidopsis thaliana* cytosolic acetoacetyl-CoA thiolase II, also known as acetyl-CoA acetyltransferase (*ACAT2*), was present at approximately 1.6 Mb downstream of FaP1A7. Moreover, an *A. thaliana* Patatin-related phospholipase A (*PLA*) gene and an *F. x ananassa* ethylene receptor (*Ers1*) gene were found 1.4 kb and 2.0 Mb downstream of FaP1D7, respectively (Fig. 3b).

The FaP1A7 was positively correlated with γ-dodecalactone production in strawberry fruits according to the differences in signal intensities between the ‘H’ (282.66) and ‘L’ (106.71) bulks (Table 4). While the precise lactone biosynthesis pathway in plants remains elusive, it is clear that the formation of γ- and δ-lactones start from β-oxidation of fatty acids^44^. Based on the fatty acid degradation pathway in the KEGG database, the acetyl-CoA acetyltransferase (*ACAT2*) gene found in close proximity to FaP1A7 is one of the thiolases that catalyses the reverse reaction in the last step of β-oxidation^45,46^.

In contrast, FaP1D7 is negatively correlated to methyl butanote based on the differences of hybridisation signal between the ‘H’ (248.23) and ‘L’ (578.61) bulks (Table 4). It has been reported that amino acids and lipids are the most likely precursors of ester formation^47^. Interestingly, the Patatin-related phospholipase A (*PLA*) strongly linked to FaP1D7 is one of the members of the phospholipase superfamily that hydrolyses the *sn1* and/or *sn2* position of the membrane phospholipid to release fatty acids^48^. The discovery of ethylene receptor (*Ers1*) at close proximity to FaP1D7 raises an interesting question regarding the role of ethylene in regulating ester formation in non-climacteric fruits, which is yet to be resolved. This result may suggest that a low amount of ethylene accumulation during strawberry fruit ripening is sufficient to regulate the ethylene receptor for the activation of phospholipase A enzyme, resulting in the release of free fatty acids as precursors for methyl ester formation^49^.

Whilst the role of the *ACAT2*, *PLA* and *Ers1* genes in controlling the levels of γ-dodecalactone and methyl butanoate in strawberry was not determined in this study, the differences in signal intensities between the ‘H’ and ‘L’ bulks may suggest the presence of allelic variants in the FaP1A7 and FaP1D7 loci. To test this hypothesis, direct amplicon sequencing was performed on the PCR product amplified from the parental genotypes, 07-102-41 and Juliette, to determine the allelic variation of these putative DNA markers.

### Detection of Single Nucleotide Polymorphism (SNP)

DNA sequence analysis obtained from FaP1D7 amplification revealed a putative C/T SNP between 07-102-41 and Juliette. By comparing to the original DNA sequence cloned into pGEM^®^-T Easy vector, the DNA fragment printed on the SDA was possibly derived from Juliette (See Supplementary Fig. S1). This result is in accordance with the SDA data, where the SNR of Juliette (i.e., 513.10) is nearly 1.4 x higher than that of 07-102-41 (i.e., 386.38) and the SNR of the ‘L’ bulk (i.e., 467.90) is almost 1.7 x higher than in the ‘H’ bulk (i.e., 268.67). This is because the DNA sequences with a ‘T’ at the SNP site would bind loosely to the SDA compared to DNA sequences carrying a ‘C’, generating an altered hybridisation pattern^50^. Based on the aroma profiles of 07-102-41 and Juliette (Table 1), higher levels of methyl butanoate were detected in 07-102-41 (15.4%) compared to Juliette (0.0%). This result suggests that the FaP1D7 marker allele harbouring either a ‘T’ or ‘C’ nucleotide at the SNP site may be associated with high or low levels of methyl butanoate production, respectively. In contrast, difficulty in interpreting the DNA sequence derived from FaP1A7 was encountered due to the overlapping of multiple peaks (chromatogram not shown), indicating the presence of secondary products even after gel purification. Hence, only FaP1D7 was selected for marker validation.

### Validation of the FaP1D7 putative marker

**Figure 4:**
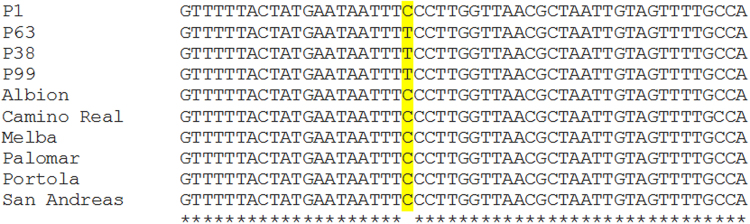
Detection of the C/T SNP in a wider range of strawberry germplasm. P1 and P63 were selected from the ‘L’ extreme bulk whereas P38 and P99 derived from the ‘H’ extreme bulk.

**Figure 5:**
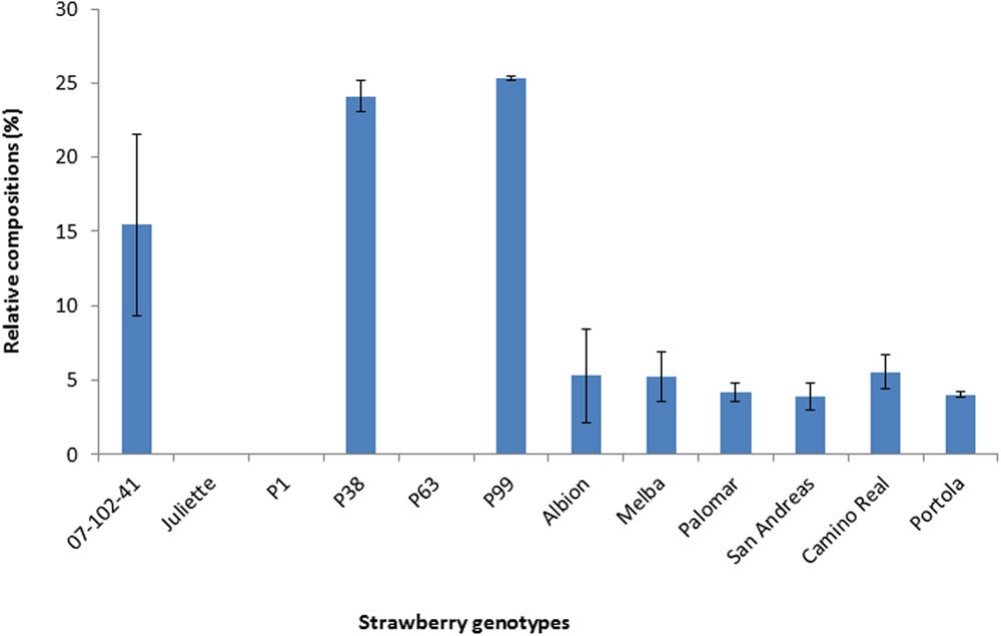
Relative compositions (%) of methyl butanoate detected in different strawberry genotypes using gas chromatography coupled with mass spectrometry. Parental genotypes: 07-102-41 and Juliette; F_1_ progeny plants: P1, P38, P63 and P99; commercial cultivars: Albion, Melba, Palomar, San Andreas, Camino Real and Portola. Error bars represent the standard deviation of the mean.

In the present study, we validated FaP1D7 putative marker for the levels of methyl butanoate accumulated in four F_1_ progeny plants exhibiting extreme phenotypes and a wider range of strawberry germplasm. DNA sequencing revealed a ‘T’ nucleotide within FaP1D7 for P38 and P99 exhibiting high levels of methyl butanoate as expected. However, a slight discrepancy was observed for the F_1_ progeny plants exhibiting low levels of methyl butanoate, where P63 showed a ‘T’ instead of ‘C’ nucleotide as in P1 (Fig. 4). DNA sequence analysis showed that all the commercial cultivars contained a ‘C’ nucleotide within FaP1D7, suggesting that these genotypes should have low levels of methyl butanoate (Fig. 4). These results are in accordance with our GC-MS analysis, where no methyl butanoate was detected in P1 and P63 whereas 24.1% and 25.3% of methyl butanoate was detected in P38 and P99, respectively. The relative composition of methyl butanoate in the commercial cultivars was relatively low at 3.9–5.6% compared to P38 and P99. The parental genotypes, 07-102-41 and Juliette are presented for comparison (Fig. 5). Our observation demonstrated expected marker patterns according to their phenotypic classification into high and low levels of methyl butanoate in all evaluated genotypes except P63. This inconsistency could possibly be due to P63 being misclassified into ‘L’ bulk due to environmental effects. Another possible explanation could be the differences in allele dosage. As the octoploid strawberries are highly heterozygous, therefore more copies of ‘T’ could be present in the genotypes producing high levels of methyl butanoate and vice versa. A similar study performed by Chambers, *et al*.^16^ also speculated that there is possibly an allele dosage effect for *NES1* allelic variants responsible for the production of linalool in octoploid wild accessions. To address this ambiguity, we propose to detect this genetic variant in different strawberry genotypes using High Resolution Melting (HRM) analysis. This may help to filter out some of the octoploid background effect. Given the data, we also propose to include cultivars or accessions producing high levels of methyl butanoate in further analysis to evaluate and strengthen the prediction power of the C/T SNP.

## Conclusions

We report the identification of a putative marker allele, FaP1D7 using an in-house developed subtracted gDNA microarray. FaP1D7 contains a C/T SNP potentially associated with the levels of methyl butanoate in strawberry fruits. While the sample sizes are quite small for validation, and in spite of the complexity of the flavour trait, there appears to be some association between phenotype and genotype. However, the results presented in this study emphasise the need of a validation in determining the effect of allele dosage of the C/T SNP. We proposed to analyse the target SNP in different strawberry germplasms with HRM analysis using real-time PCR. More strawberry genotypes and/or crosses are required to confirm the functionality of the C/T SNP potentially associated with the levels of methyl butanote in strawberry fruits.

## Methods

### Parental genotypes and segregating population

The idea behind the project is to incorporate unique strawberry aroma from selected breeding lines into one of the Australian cultivars. Juliette, a short day cultivar bred by the Victorian Department of Primary Industries (DPI), is selected as parental genotype because it produces fruits that are sweeter than other cultivars developed through the same breeding program. It is widely accepted by consumers^51^. It is a bright red strawberry which fruits in the early season (September) in Victoria, Australia. Three promising breeding lines, including 07-102-41, 07-095-35 and 04-069-91 were considered as parental genotypes. Of these, 07-102-41 which has a primarily European genetic background was selected for crossing because it produces unique and very flavourful fruits. It is a short day strawberry with dark red fruits, and genetically diverse from Juliette. The segregating population consisted of 200 F_1_ plants derived from a cross between ‘Juliette’ and ‘07-102-41’. A subset of 50 F_1_ individuals was randomly selected as experimental materials based on the healthiness of the plants and the availability of firm fruits per plant. Of these, 37 progeny plants had 07-102-41 as a maternal parent (07-102-41 × Juliette) and the remaining 13 progeny plants were collected from the reciprocal cross (Juliette × 07-102-41) where Juliette was used as the maternal parent. Fully ripe fruits and young leaves from both parental genotypes and F_1_ population were harvested over the summer of 2011/2012 for aroma profiling and DNA genotyping, respectively. Similarly, fruits and leaves of six commercial cultivars including Melba, Camino Real, Portola, Palomar, San Andreas and Albion were collected across 16 weeks (13/11/2013 – 9/4/2014) for marker validation.

### Profiling of strawberry aroma

#### Extraction of aroma compounds

Aroma compounds were extracted from the fruit puree using Solid Phase Microextraction (SPME) method^52^. Five large berries per plant were thawed and homogenised with a portable blender. Approximately 1 g of puree was immediately dispensed into individual SPME vials with screw caps and stored at −80 °C. Prior to gas chromatography-mass spectrometry (GC-MS) analysis, the sample was thawed to room temperature for 20 min and pre-equilibrated at 60 °C in a heating block for 10 min. The aroma compounds were extracted using a 65 µm polydimethylxiloxane/divinylbenzene (PDMS/DVB)-coated fiber held in an SPME Holder 57330-U (Supelco, Bellafonte, PA, USA). This fiber was first conditioned at 250 °C for 30 min, and then exposed to the vial headspace for 30 min at 60 °C. After equilibrium, the fiber was removed from the sample and the analytes were thermally desorbed in a GC injector port at 250 °C for 3 min. Aroma profiling for each sample was performed in triplicate.

#### GC-MS

Aroma profiling for each sample was performed in triplicate using Agilent 6890 GC coupled with a 5973 MS detector (Agilent, CA, USA) through a heated transfer line at 280 °C. Compounds were separated using DB-5ms column with dimensions of 30 m × 0.25 mm I.D. × 0.25 µm film thickness. Helium was used as a carrier gas at a flow rate of 1.5 mL/min. 1.0 µL was injected using the splitless injection mode with a 2.5 min of solvent delay. The oven temperature was programmed initially at 40 °C for 1 min, then increased at a rate of 6 °C/min to 190 °C and kept constant at the same temperature for 26 min with a final isotherm at 190 °C for 4 min. The MS source temperature was 230 °C and the compounds were monitored over the mass range m/z 45–400.

#### GC-MS data analysis

A similarity search was carried out by comparing the retention times and quality of known compounds in Wiley and Adams mass spectra libraries. All the chromatographic peaks found in two or more technical replicates of the same sample and with a quality greater than 80 were taken into account. The relative composition of aroma compounds (%) in the headspace of the strawberry puree was quantitated based on area normalisation method with two assumptions: (1) detector response is the same for different compounds, and (2) compounds of the sample injected are completely detected and will produce peaks^53^. The calculation was done according to the equation below:

1 $${{\rm{C}}}_{{\rm{i}}}={{\rm{A}}}_{{\rm{i}}}/{{\rm{A}}}_{{\rm{t}}}\times {\rm{100}} \% $$

where C_i_ = Content of a compound in the sample.

Ai = Area of compound peak in the chromatogram.

At = Total area of the peaks in the chromatogram.

The mean relative composition and standard deviation of each compound were calculated from three technical replicates. Aroma compounds detected by GC-MS and their relative compositions were categorised according to different chemical groups. Target compounds for DNA marker development were chosen based on the distinctive parental aroma profiles and their relative contribution to strawberry flavour as elucidated in a number of publications^5,6,9,13,30,54^. Application of these selection criteria resulted in the selection of eight compounds of interest including four esters (methyl butanoate, methyl hexanoate, ethyl butanoate and ethyl hexanoate), one furanone (mesifuranne), two terpenes (linalool and (*E*)-nerolidol) and one lactone (γ-dodecalactone).

### Generation of DNA bulks with extreme phenotypes

The number of plants with extreme phenotypes for the selected compounds was identified by generating frequency distributions from the 50 F_1_ progeny plants along with their parental means using Microsoft Excel. The F_1_ plants determined from the segregation patterns were selected for BSA. Total genomic DNA was isolated from the leaves of individual plants using Qiagen^TM^ DNeasy^®^ Plant Mini Kit (Qiagen, Valencia, CA) according to manufacturer’s instructions. Equal amounts of DNA from F_1_ progeny plants showing high (H) or low/undetectable (L) levels of key aroma compounds were pooled into the respective ‘H’ and ‘L’ bulks to a final quantity of 2 µg. The number of individuals in each bulk ranged from 3 to 27 plants depending on the key aroma compounds.

### Microarray-assisted Bulked Segregation Analysis

A 287-feature *Fragaria* Discovery Panel (FDP) constructed and validated previously was used as a platform for screening of molecular markers associated with key aroma compounds^24^. Marker discovery was performed by hybridising 16 DNA bulks corresponding to the ‘H’ and ‘L’ extremes of eight key aroma compounds onto the FDP. Target DNA was labelled with Biotin-11-dUTP molecules as described by Gor, *et al*.^24^. Hybridisation of the biotinylated DNA targets onto the FDP and fluorescent detection using a biotin-streptavidin system was performed according to Mantri, *et al*.^55^. All hybridisations were performed with six technical replicates and two biological replicates to ensure microarray reproducibility, producing a total of 12 data points per feature for subsequent statistical analysis. FDP slide scanning, microarray image capturing and data normalisation were performed based on Gor, *et al*.^24^. All microarray experiments were compliant with MIAME guidelines and all data have been deposited in Gene Expression Omnibus (GSE70145).

### Statistical analysis

The FDP data was subjected to Discriminant Function Analysis (DFA) to identify molecular markers associated with fruit flavour. The ‘H’ and ‘L’ phenotypic groups and the normalised mean SNR of the 287 FDP features were used as dependent and independent variables, respectively. DFA was performed using IBM SPSS Statistics v. 21 with a stepwise method for the selection of the most discriminative features between the two phenotypic extreme groups. Wilks’ lambda was used to determine the classification efficiency of each feature based on the default *F* probability values (Entry = 0.05, Removal = 0.10). In this study, six technical replicates (original cases) from the first biological replicate of a phenotypic group were assigned as a training set to predict the group membership of the other six technical replicates (new cases in the test set) from the second biological replicate. All the twelve technical replicates of the selected features were subjected to stepwise analysis again to form classification models using Fisher’s classification function coefficients. The performance of the discriminant function was evaluated using the cross-validation method.

For validation of the DFA-selected features, Fisher’s ratio was employed and calculated according to Lohninger^56^:

2 $$\mathrm{Fisher}\mbox{'}s\,{\rm{ratio}}={({{\rm{M}}}_{1}-{{\rm{M}}}_{2})}^{2}/({{\rm{V}}}_{1}+{{\rm{V}}}_{2})$$

where M_1_ = Mean of the normalised SNR for each feature in the ‘H’ bulk, M_2_ = Mean of the normalised SNR for each feature in the ‘L’ bulk, V_1_ = Variance of the normalised SNR for each feature in the ‘H’ bulk and V_2_ = Variance of the normalised SNR for each feature in the ‘L’ bulk.

Independent Samples *t*-Test (IBM SPSS Statistics v. 21) was performed using all the twelve technical replicates of the ‘H’ and ‘L’ bulks as variables. Only the features showing high Fisher’s ratio (top 10) and significant differences between the group means of ‘H’ and ‘L’ bulks (*p* < 0.01) were retained for further analysis. Finally, a three-way Venn diagram was generated (http://www.pangloss.com/seidel/Protocols/venn.cgi) to identify putative DNA markers that fulfilled all three selection criteria.

### DNA sequence analysis

#### DNA sequencing of putative molecular markers

Plasmids corresponding to the putative DNA markers were sequenced bi-directionally at Macrogen Inc. (Korea) using T7 and Sp6 primers. Similarity search was performed against the *Fragaria vesca* draft genome (v1.1) using PFR Strawberry Server (https://strawberry.plantandfood.co.nz/) and confirmed with Genome Database for Rosaceae (http://www.rosaceae.org/tools/ncbi_blast). Sequence identity with an E-value < 1e^−5^ was considered significant. Subsequently, genes located within 5 centiMorgan (cM) on either side of the putative DNA markers were manually searched using PFR Strawberry Server based on previously mapped genes available in Strawberry Genbank and general RefSeq mRNA database. By assuming the genetic length of a normal chromosome as 100 cM^57^, the physical distance covering 5 cM was calculated following the equation below:

3 $${\rm{Physical}}\,{\rm{distance}}\,({\rm{Mb}})=(\mathrm{Physical}\,{\rm{length}}\,{\rm{of}}\,{\rm{the}}\,{\rm{linkage}}\,\mathrm{group}/\mathrm{100}\,\mathrm{cM})\times {\rm{5}}\,{\rm{cM}}$$

#### Determination of DNA sequence polymorphism

DNA sequences showing significant similarity (E-value < 1e^−5^) to *F. vesca* nuclear sequences were chosen for primer design using Clone Manager Suite v. 7.1 (Sci-Ed Software, Durham, NC). To determine the DNA microstructural variation of the putative markers between the ‘H’ and ‘L’ DNA bulks, PCR amplification was performed on both the parental genotypes (Juliette and 07-102-41) using AccuPrime™ *Pfx* DNA Polymerase (Invitrogen, NY, USA). Briefly, 50 ng of genomic DNA was used as template in a 50 µL PCR reaction containing 1 X of AccuPrime™ *Pfx* mix, 40 nM of each sequence-specific forward and reverse primer, 1 U of AccuPrime™ *Pfx* DNA Polymerase and water. The thermal cycling conditions were as follows: initial denaturation at 95 °C for 2 min; followed by 30 cycles of denaturation at 95 °C for 15 s, annealing at 55 °C for 30 s, extension at 68 °C for 1 min. The integrity and length of PCR products were examined using 2.0% TBE agarose gel electrophoresis. The PCR products were subsequently purified using Qiaquick Gel Extraction Kit (Qiagen, Valencia, CA) and sequenced by Australian Genome Research Facility Ltd. (AGRF) using the sequence-specific primers. All the forward and reverse DNA sequences were aligned for using the Clustal Omega Multiple Sequence Alignment function at https://www.ebi.ac.uk/Tools/msa/clustalo/ to identify any SNPs or indels within the marker. All sequences were deposited in NCBI GeneBank database (KT162989 – KT163008).

### Validation of the putative molecular marker

The putative molecular marker that showed sequence polymorphism between the parental genotypes was selected for validation in the F_1_ segregating population and a wider germplasm. For the purpose of this study, two high level of methyl butanoate producing F_1_ progeny (P38 and P99) and two low levels of methyl butanoate producing F_1_ progeny (P1 and P63) along with six commercial cultivars including Melba, Camino Real, Portola, Palomar, San Andreas and Albion were selected for marker validation. The aroma profile for these six commercial cultivars was evaluated using GC-MS as described above. The putative marker sequence was amplified from these plants, cloned into a pGEM-T vector and transformed into *E. coli* JM109. 10 of the colonies were randomly picked for each genotype and the clones were sequenced bidirectionally using T7 and SP6 primers. The resulting sequences were aligned with sequences derived from the parental genotypes to deduce the association between the polymorphic markers and key aroma compounds.

## Electronic supplementary material

Supplementary Figure S1
