# Supplementary material for: Identification and validation of FaP1D7, a putative marker associated with the biosynthesis of methyl butanoate in cultivated strawberry (Fragaria x ananassa)
Source: Sci Rep. 2017 Dec 12;7:17454. doi: 10.1038/s41598-017-17448-1 (PMC5727213; doi:10.1038/s41598-017-17448-1)
Supplement: Supplementary file 1 — Supplementary Figure S1 [file 41598_2017_17448_MOESM1_ESM.pdf]

Identification and validation of FaP1D7, a putative marker associated with the biosynthesis of methyl butanoate in cultivated strawberry (*Fragaria x ananassa*)

Mian Chee Gor[1,2], Chrishani Candappa[1], Thishakya de Silva[1], Nitin Mantri [1*], Edwin Pang[1]

[1]School of Science, RMIT University, Plenty Road, PO Box 71, Bundoora, Victoria. 3083 Australia
[2]Griffith Institute for Drug Discovery (GRIDD), Don Young Road, Nathan, Queensland, 4122 Australia.

*Correspondence (telephone +61 3 99257597; fax +61 3 99257110; nitin.mantri@rmit.edu.au)

```
FLP1D7       AAATGATACCTTGGGACGGATGTAAAGCTAAGCAAATCGCTTCTGCTCCAAGAACAGAGG
07-102-41    ----------------------------------AATCGCTTCTGCTCCAAGAACAGAGG
Juliette     ----------------------------------AATCGCTTCTGCTCCAAGAACAGAGG
                                               **************************

FLP1D7       ACTGTGTCGGTTGTAAGAGATGTGAATCCGCCTGTCCAACGGATTTCTTGAGTGTTCGCG
07-102-41    ACTGTGTCGGTTGTAAGAGATGTGAATCCGCCTGTCCAACGGATTTCTTGAGTGTTCGCG
Juliette     ACTGTGTCGGTTGTAAGAGATGTGAATCCGCCTGTCCAACGGATTTCTTGAGTGTTCGCG
             ************************************************************

FLP1D7       TTTATTTATGGCATGAAACAACTCGCAGTATGGGTCTAGCTTATTAATACGTTCCAGAAA
07-102-41    TTTATTTATGGCATGAAACAACTCGCAGTATGGGTCTAGCTTATTAATACGTTCCAGAAA
Juliette     TTTATTTATGGCATGAAACAACTCGCAGTATGGGTCTAGCTTATTAATACGTTCCAGAAA
             ************************************************************

FLP1D7       ACCCTACTCGAATACATTTGATTTTTTTACCTTTATCGACAAAAACCCGCGCTCAAAATA
07-102-41    ACCCTACTCGAATACATTTGATTTTTTTACCTTTATCGACAAAAACCCGCGCTCAAAATA
Juliette     ACCCTACTCGAATACATTTGATTTTTTTACCTTTATCGACAAAAACCCGCGCTCAAAATA
             ************************************************************

FLP1D7       TATTTATTTCGAGCACGGGTTTTTCTGGTCCAAGTTTATTTTGTTTTTACTATGAATAAT
07-102-41    TATTTATTTCGAGCACGGGTTTTTCTGGTCCAAGTTTATTTTGTTTTTACTATGAATAAT
Juliette     TATTTATTTCGAGCACGGGTTTTTCTGGTCCAAGTTTATTTTGTTTTTACTATGAATAAT
             ************************************************************

FLP1D7       TTCCCTTGGTTAACGCTAATTGTAGTTTTGCCAATATCCGCGGGTTCCTTAATTTT
07-102-41    TTTCCTTGGTTAACGCTAATTGTAGTTTTGCCAAT---------------------
Juliette     TTCCCTTGGTTAACGCTAATTGTAGTTTTGCCA----------------------
             ** *******************************
```

a

07-102-41

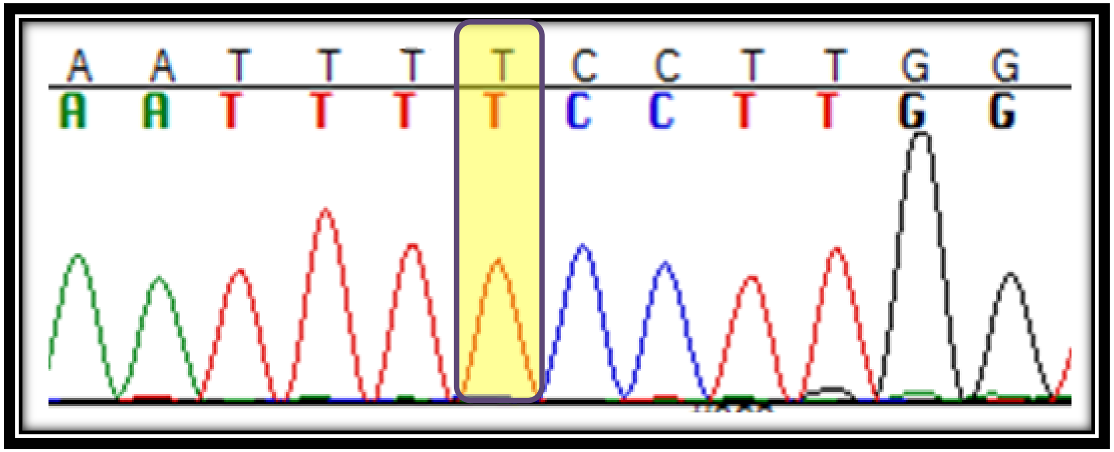

Juliette

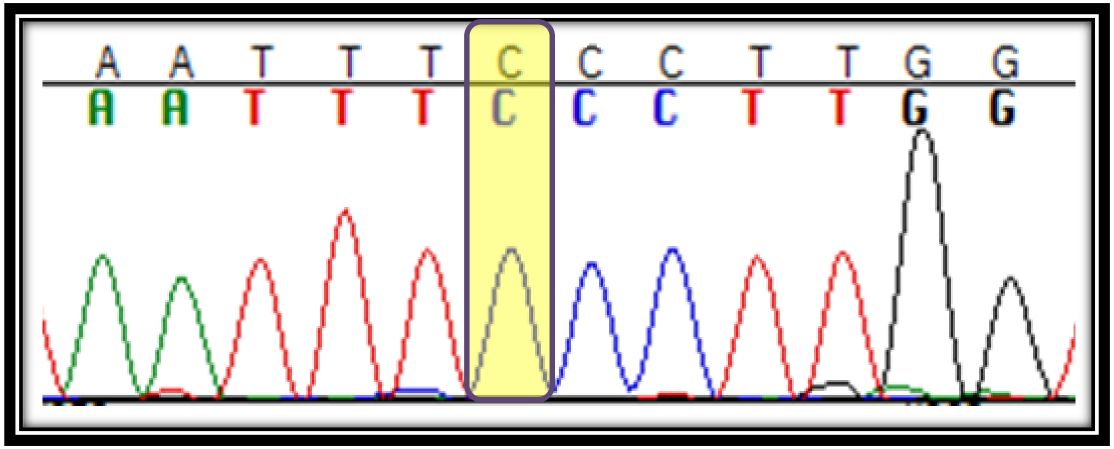

b

Supplementary Figure S1. DNA sequence polymorphism between two parental genotypes. (a) DNA sequence alignment of PCR products amplified from 07-102-41 and Juliette using FaP1D7-specific primers. The original FaP1D7 derived from pGEM®-T Easy vector was used as a reference sequence. (b) Chromatograms showing the putative C/T SNP between 07-102-41 and Juliette. The C/T SNP is highlighted in yellow.
